# Thalamo-Habenular Connection Differences Between Patients With Major Depressive Disorder and Normal Controls

**DOI:** 10.3389/fpsyt.2021.699416

**Published:** 2021-09-01

**Authors:** Seo-Eun Cho, Nambeom Kim, Kyoung-Sae Na, Chang-Ki Kang, Seung-Gul Kang

**Affiliations:** ^1^Department of Psychiatry, Gil Medical Center, Gachon University College of Medicine, Incheon, South Korea; ^2^Department of Biomedical Engineering Research Center, Gachon University, Incheon, South Korea; ^3^Department of Radiological Science, College of Health Science, Gachon University, Incheon, South Korea

**Keywords:** diffusion tensor imaging, major depressive disorder, habenula, thalamus, fiber connection

## Abstract

**Background:** The thalamus and habenula are thought to be key brain regions in the etiology of major depressive disorder (MDD); however, few studies have investigated the structural connection between them. We compared the number of white matter tracts between the thalamus and habenula between patient with MDD and normal controls (NCs).

**Methods:** The habenula and thalamus region of interest masks were extracted from brain magnetic resonance imaging data and individual tractography analysis was performed. First, we compared the number of fiber connections from the habenula to the thalamus between the MDD (*n* = 34) and NC (*n* = 37) groups and also compared hemispherical differences to investigate possible asymmetries.

**Results:** There was a significant difference in the number of tracts in the right habenula-left mediodorsal thalamus pair between the two groups. For hemispherical fiber connections, the waytotal ratio of the right ipsilateral tract between the thalamus and habenula was significantly higher than that of the left ipsilateral tract in both groups.

**Conclusion:** The number of right habenula-left mediodorsal thalamus tracts was higher in patients with MDD than in NCs. These results indicate that MDD is related to the disintegration of the left thalamus-right habenula tract function with an increased number of tracts as a compensational mechanism.

## Introduction

Major depressive disorder (MDD) is one of the most prevalent and disabling psychiatric disorders (1), and it has led to an increase in healthcare utilization and consequent social burden and economic cost (2). MDD is the leading cause of severe functional impairment and poor quality of life due to symptoms such as depressed mood, loss of interest, sleep and appetite changes, hopelessness, feelings of guilt, and suicidal ideation lasting longer than two weeks (3–5). Therefore, MDD is a heterogeneous disorder in which several symptoms and signs appear due to various causes and include diverse neural tracts (6, 7).

The thalamus is considered to be one of the major brain regions involved in the pathophysiology of depression, emotions, and restorative autonomic and endocrine processes (8). Previous voxel-based morphometry studies have shown smaller thalamic volumes in patients with MDD than in normal controls (NCs) (9–11). In addition, there was a significant difference in the volume of the bilateral thalami using an automatic segmentation tool between patients with MDD and NCs after controlling for total intracranial volume (12). Meanwhile, the thalamus showed increased metabolism in a positron emission tomography study (13), and significantly greater connectivity with the default-mode network in a resting state functional magnetic resonance imaging (MRI) study (14).

Since the detailed anatomy of the habenula was revealed by Marburg (15), it has been reported that this area plays an important role in the stress response and is linked to brain areas related to emotion and behavior (16, 17). Animal studies have implicated the lateral habenula as a key brain region in the pathophysiology of major depression (18). In previous studies, the habenula was activated by aversive emotional stimuli or negative reward prediction errors and the activation was subsequently related to the suppression of the monoaminergic nuclei, including the ventral tegmental area, locus coeruleus, and dorsal raphe, which are known to release important neurotransmitters in depression (18, 19). The habenula is also involved in emotional and cognitive processes, having connections to many other areas of the brain (e.g., thalamus, prefrontal cortex, basal ganglia, and brainstem monoaminergic neurotransmitter systems) (16, 20). Among them, thalamus-habenula connectivity has been suggested to play an important role in neurobiological models of MDD (18). A recent brain imaging study of connectome-based biomarkers using machine learning has shown that subclinical depression is associated with abnormal brain connections in the subregions of the thalamus and lateral habenula (21). The habenula is primarily connected to other areas of the brain through the stria medullaris and fasciculus retroflexus (22). The stria medullaris is connected to areas of the telencephalon such as the thalamus, caudate, putamen, globus pallidus, amygdala, and hippocampus and projects to the habenula. The fasciculus retroflexus is connected to monoaminergic systems such as the ventral tegmental area, substantia nigra, and dorsal raphe nuclei (23, 24). Previous studies have reported that the stria medullaris, one of the main afferent tracts of the habenula and connected with the thalamus, is important for the pathophysiology of depression (24). In addition, deep brain stimulation of the stria medullaris was reported to be an effective treatment in patients with treatment-resistant depression (25–27).

Despite the evidence of the importance of these two brain areas (the thalamus and habenula) in depression and that a number of brain imaging studies have been conducted to elucidate the etiology of MDD, the link between these two brain areas has not been studied in clinical subjects. In particular, there has been no study using diffusion tensor imaging (DTI), which allows visualization of white matter integrity, to examine this tract in patients with MDD. A white matter tract is a bundle of parallel myelinated axons, and diffusion-weighted MRI can investigate microscopic changes that are related to myelin, cellular organelles, and the movement of water molecules in the white matter (28). Therefore, a study of whether the tract linking the thalamus and habenula differs between patients with MDD and NCs will be important in revealing the etiology of MDD.

Asymmetrical structural circuits of the habenula have been found in vertebrates in previous studies and experiments (29). Various species, including fish and amphibians, exhibit left–right asymmetry of neuronal differentiation (29). In humans, a previous study reported that the left lateral habenula was larger than the right habenula by ~5% (30), and left–right asymmetry of habenula volume in MDD was also found in a 7T brain MRI study conducted by our research team (31). In addition, the asymmetry of functional connections of the human habenula with monoamine centers (i.e., substantia nigra and ventral tegmental area) has been reported in previous studies (32). Therefore, studies on whether the thalamo-habenula tract exhibits structural or functional asymmetry in depression would be of great interest.

The aims of this study were (1) to investigate whether the number of tracts from the habenula to the thalamus differs between patients with MDD and NCs, and (2) to investigate the difference in the waytotal ratio (WTr) of the ipsilateral tracts from the habenula to the mediodorsal thalamus between the left and right hemispheres in the MDD and NC groups.

## Methods

### Participants and Clinical Measurement

We recruited participants from the Department of Psychiatry, Gil Medical Center, Incheon, South Korea in two groups: NCs and patients with MDD. All participants provided written informed consent to participate in the study. This study was approved by the Institutional Review Board (IRB No. GDIRB2018-005) of the Gil Medical Center. The board-certified psychiatrist assessed the participants' eligibility for this study using a Structured Clinical Interview for the fifth edition of the Diagnostic and Statistical Manual of Mental Disorders (DSM-5) (33). Patients meeting the DSM-5 diagnostic criteria for MDD were included in the MDD group (5). Furthermore, participants in the MDD group did not have any of the following psychiatric comorbidities: schizophrenia spectrum and other psychotic disorders, major anxiety disorders, obsessive-compulsive disorder, or disruptive, impulse-control, and conduct disorders. Depression severity was quantified using the HDRS-17 (34), CGI-S (35), and BDI (36, 37).

The following common exclusion criteria were applied: age under 20 or over 65 years, left-handed using the Edinburgh Handedness Test, unstable or major medical condition, neurological disorders within the past 1 year, substance abuse within the past 1 year, intellectual disability, neurocognitive disorders, personality disorder, high risk of suicide, history of head trauma, previous abnormal findings in brain imaging, contraindications to MRI (e.g., metals or electronic devices in the body), and pregnancy or lactation. Additional exclusion criteria were added for NCs: psychiatric history, HDRS-17 total score > 6, history of taking psychotropic medications, and first-degree relatives with major psychiatric disorders such as schizophrenia, MDD, or bipolar disorders.

### Image Acquisition

We acquired the whole-brain images using a 12-channel phase-array coil at 3T MRI (MAGNETOM 3T, Siemens Verio, Erlangen, Germany). A pillow was placed under the participants' head for a comfortable position and foam cushions were used to minimize head movement. A spin-echo echo planar imaging (EPI) sequence was used for DTI with the following acquisition parameters: diffusion gradients applied in 30 directions with *b*-values of 0 and 900 s/mm^2^ (38); field of view = 230 × 230 × 144 mm^3^; matrix size = 128 × 128, 80 slices with no gap; resolution of 1.8 × 1.8 × 1.8 mm^3^; repetition time (TR) = 13,100 ms; echo time (TE) = 78 ms; pixel bandwidth = 1,502 Hz/px; scan time (TA) = 14 min 39 s; accelerating factor using generalized auto-calibrating partially parallel acquisitions (GRAPPA) = 3 (60 auto-calibration signal lines); and 6/8 partial Fourier factors along the phase-encoding.

In addition to the DTI, a three-dimensional (3D) magnetization-prepared rapid gradient-echo (MPRAGE) sequence was used for high-resolution T1-weighted images of all the participants with the following acquisition parameters: field of view = 208 × 256 × 160; isotropic resolution of 1.00 mm^3^; matrix size = 208 × 256, 160 slices; TR = 1,900 ms; TE = 3.3 ms; inversion time = 900 ms; flip angle = 9°; pixel bandwidth = 140 Hz/px; TA = 3 min 40 s; accelerating factor = 2 (24 auto-calibration signal lines); and 7/8 partial Fourier factors along the phase-encoding.

### Tractography

**Figure 1:**
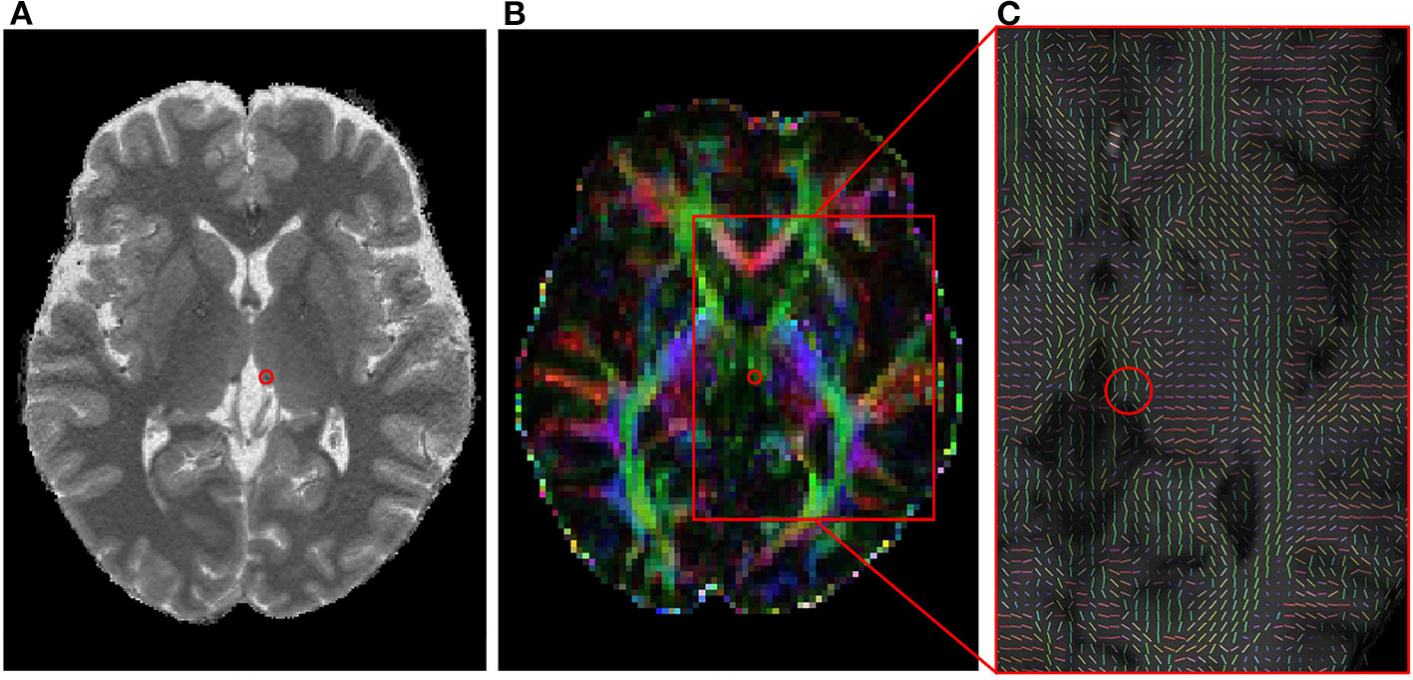
The habenula in a 3T MRI image and its diffusion tensor image. **(A)** A selected T1 image indicating a habenula area with a red circle. **(B)** A fractional anisotropy image corresponding to **(A)**. **(C)** A zoomed fiber orientation image estimated with BEDPOSTX. The mean principle diffusion direction distribution in the red box of **(B)** was shown in a vector form. MRI, magnetic resonance imaging.

DTI data of each participant were individually analyzed using the Oxford Center for FMRIB Diffusion Toolbox of Functional MRI of the Brain (FMRIB) software library (FSL ver. 5.0.9) (http://www.fmrib.ox.ac.uk/fsl). The data in DICOM format of each participant were first converted into a file format usable in FSL using dcm2nii (https://www.nitrc.org/projects/dcm2nii/), and then analyzed according to the FSL standard procedures (39). The skull was removed in the baseline non-diffusion weighted (b0) image to create a mask image, and head motion and eddy current distribution were corrected. Then, the Bayesian Estimation of Diffusion Parameters Obtained Using Sampling Techniques (BEDPOSTX) tool in FSL was used to build up distributions on diffusion parameters and model the crossing fibers within each voxel, which were then fitted to the mask image (Figure 1). Finally, the diffusion images were pre-processed using a linear registration (FLIRT) on the anatomical image and non-linear registration (FNIRT) on the Montreal Neurological Institute (MNI152) template for probabilistic tractography.

To examine the fiber connections between the habenula and thalamus, region of interest (ROI) mask images were created prior to the tractography processing. The diffusion space of each participant and the ROI mask segmented from the MNI template must be in the same standard space; thus, linear and nonlinear transformation matrices were generated during the registration process and then utilized together. The habenula was manually segmented by an experienced researcher on the spatially normalized single-subject high-resolution T1 volume image (40, 41) using MRIcron (http://www.mccauslandcenter.sc.edu/mricro/mricron/); the segmented result was then confirmed by another senior researcher (see Supplementary Figure 1 for the segmented masks overlaid on the T1 template). The thalamic area from the automated anatomical labeling atlas 3 (AAL3) template was used (42). Based on the results of previous studies (43, 44), three regions of the thalamus, the anteroventral thalamus (Thal_AV), ventral anterior thalamus (Thal_VA), and magnocellular portion of the mediodorsal nucleus of the thalamus (Thal_MDm), were selected as ROIs and analyzed.

Probabilistic tractography was obtained using FSL's probtrackx2 tool where the habenula mask was set as the seed ROI and was performed in a single mask mode with the parameters of 5,000 sample pathways that can be created in one voxel and a 0.2 curvature threshold. Additionally, by setting the waypoint mask and the terminal mask in the thalamus, which was the target ROI, the direct connections between the seed ROI and the target ROI were calculated.

For each analysis, a streamline density map was created along with the WT, which is the number of tracts directly connected between the seed and target ROIs. The results were displayed using FSLeyes version 0.34.2 (https://fsl.fmrib.ox.ac.uk/fsl/fslwiki/FSLeyes). The WTr for the connections between the habenula and thalamus of each participant was also obtained, where the WTr of each connection pair was the percentage of the total tracts that can be generated at the seed ROI, that is, WTr = WT/total tracts × 100, where total tracts is the total number of seed voxels × 5,000 sample pathways.

### Statistics

The distribution of data was presented as mean and standard deviation for parametric analysis and mean, standard deviation, median, and interquartile range for nonparametric analysis. Demographic data and clinical characteristics, including the clinical scales of the HDRS-17, BDI, and CGI were compared between the two groups using a Student's *t*-test. The chi-square test was used for categorical and ordinal variables (sex and CGI-S). For DTI data analysis, the normality of the data was assessed with a Kolmogorov–Smirnov test before performing a parametric test. The group difference of probabilistic tractography between the MDD and NC groups was performed using the Mann–Whitney *U*-test. In this analysis, the Bonferroni-adjusted significance level was set as *p* < 0.0083 (*p*-value/6) since the number of ROIs in the thalamus was 6. Furthermore, to investigate the correlation between clinical characteristics (i.e., education period, duration of illness, duration of antidepressant use, and HDRS-17 total score) and thalamo-habenular tracts with significant differences in the number of tracts between groups, partial correlation analysis controlling for age was performed in the MDD group. The mean difference between the left and right hemispheres in each group was analyzed using a paired *t*-test. All statistical analyses were performed using SPSS v21.0 (IBM Corp., Armonk, NY).

## Results

### Demographics and Clinical Characteristics

A total of 71 participants (34 patients with MDD and 37 NCs) were included in the analysis. The demographic and clinical characteristics of the participants are presented in Table 1. The NC and MDD patient groups did not significantly differ in age or in the proportion of females. The mean duration of illness in the MDD group was 5.29 years. On the clinical scales, patients with MDD had significantly higher Hamilton Depression Rating Scale (HDRS, *p* < 0.001), Beck Depression Inventory (BDI, *p* < 0.001), and Clinical Global Impression-Severity (CGI-S, *p* < 0.001) scores than did the NCs. Seventy-nine percent of the MDD group were taking antidepressants, and the average duration of antidepressant use was 20.55 months (Table 1).

**Table 1:** Comparison of demographic and clinical information between the MDD and NC groups.

**Variables**	**MDD (*n* = 34)**	**NC (*n* = 37)**	Statistical tests^*^		
			***t* or *x^**2**^***	***df***	***p***
Age, years	40.71 ± 13.97	35.65 ± 12.19	*t* = 1.628	69	0.108^a^
Sex, female	26 (76.5)	25 (67.6)	*x^2^* = 0.694	1	0.405^b^
Education, years (mean ± SD)	12.79 ± 3.42	15.14 ± 1.96	*t* = 3.576	69	0.001^a^
Duration of illness, years	5.29 ± 4.82	N/A	N/A	N/A	N/A
Recurrent episode	20 (58.8)	N/A	N/A	N/A	N/A
**Clinical scales**					
HDRS-17	15.50 ± 5.70	2.62 ± 2.44	*t* = 12.195	43.942	<0.001^a^
BDI	27.06 ± 13.17	3.62 ± 3.70	*t* = 10.016	37.774	<0.001^a^
CGI-S	3.97 ± 0.99	1.05 ± 0.23	*x^2^*= 65.657	4	<0.001^b^
Number of subjects taking antidepressants (%)	27 (79)	0	N/A	N/A	N/A
Duration of antidepressants, weeks	82.18 ± 26.24	N/A	N/A	N/A	N/A

*BDI, Beck Depression Inventory; CGI-S, Clinical Global Impression-Severity; HDRS-17, 17-item version of the Hamilton Depression Rating Scale; MDD, major depressive disorder; NC, normal control*.

* *Statistical tests were performed using*

a *Student's t-test or*

b *chi-square test. Data are presented as means ± standard deviations or numbers (percentages)*.

### Comparison of the Number of Tracts From the Habenula to the Thalamus Between the MDD and NC Groups

**Figure 2:**
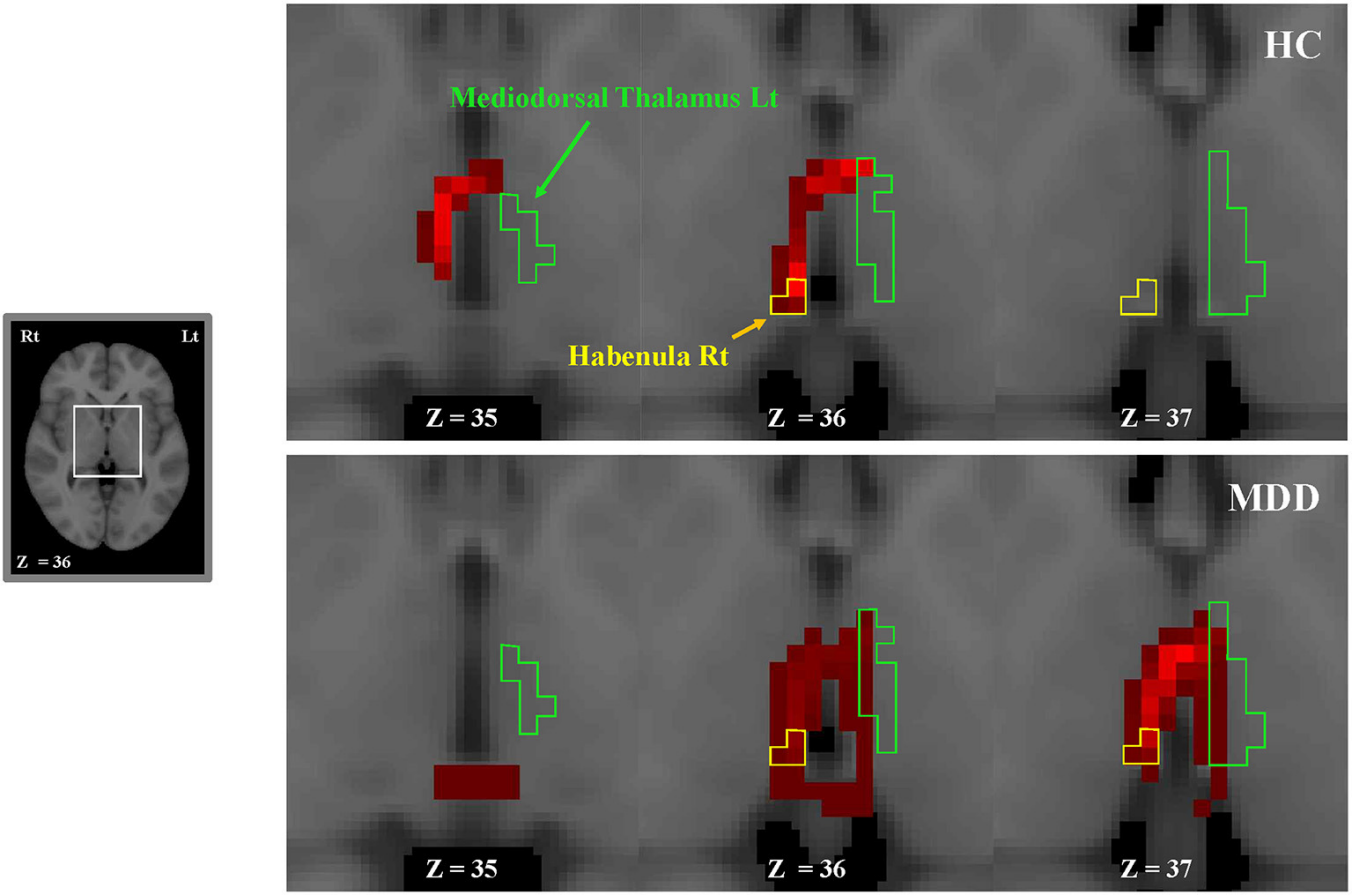
Consecutive tractography images in a representative MDD (bottom) and NC (top) participant. The tracts extend from the left or right habenula to the left or right thalamus (ipsilaterally or contralaterally). More fiber connections between the right habenula and the left mediodorsal thalamus are shown in the MDD group than in NCs. We visualized fiber connections between the right habenula and the left mediodorsal thalamus in red. The green and yellow boundaries represent regions of the left thalamus and the right habenula, respectively. Lt, left; MDD, major depressive disorder; NC, normal control; Rt, right.

The number of direct tracts (i.e., waytotal; WT) from the habenula to the thalamus was compared between the MDD and NC groups. There was a significant difference in the number of tracts only in the right habenula-left mediodorsal thalamus pair between the two groups (MDD, 113.8 ± 309.6 [mean ± standard deviation]; NC, 35.2 ± 128.7; *p* = 0.004; Table 2). However, there was no significant difference in the other pairs (i.e., right habenula-right mediodorsal thalamus, left habenula-left mediodorsal thalamus, and left habenula-right mediodorsal thalamus). This result (more fiber connections in the right habenula-left mediodorsal thalamus pair in patients with MDD than in NCs) is also clearly shown in tractography images (Figure 2).

**Table 2:** Comparison of the number of tracts from the habenula to the thalamus between patients with MDD and NCs.

**Seed ROI**	**Target ROI**	**MDD**		**NC**		**Statistics**	
		**Median** **(IQR)**	**Mean ±** **SD**	**Median** **(IQR)**	**Mean ±** **SD**	**Mann–** **Whitney *U***	***p***
Habenula_Lt	Thal_AV_Lt	20.5 (53.0)	110.0 ± 203.4	25.0 (70.0)	241.7 ± 875.0	574	0.530
	Thal_AV_Rt	5.0 (85.0)	67.9 ± 123.9	20.0 (64.0)	46.1 ± 61.1	543	0.322
	Thal_VA_Lt	55.0 (209.0)	286.8 ± 502.9	113.0 (390.0)	473.8 ± 1,468.9	595	0.700
	Thal_VA_Rt	19.5 (134.8)	138.3 ± 285.6	17.0 (64.0)	96.3 ± 234.7	586	0.625
	Thal_MDm_Lt	25,376.0 (3,654.8)	25,817.3 ± 3,319.5	24,549.0 (3,531.0)	24,984.0 ± 2,997.8	526	0.240
	Thal_MDm_Rt	2,278.0 (2,184.3)	2,810.0 ± 2,168.9	1,980.0 (2,649.0)	2,536.5 ± 2,094.5	574	0.533
Habenula_Rt	Thal_AV_Lt	0.0 (1.0)	1.0 ± 2.7	0.0 (0.0)	0.9 ± 4.7	534	0.127
	Thal_AV_Rt	48.5 (142.0)	136.9 ± 244.8	77.0 (113.0)	163.2 ± 274.1	622	0.936
	Thal_VA_Lt	0.0 (1.0)	2.1 ± 6.9	0.0 (0.0)	1.8 ± 8.1	565	0.328
	Thal_VA_Rt	1,352.0 (1,651.0)	1,916.0 ± 1,696.6	1,660.0 (3,155.0)	2,238.6 ± 1,854.6	575	0.534
	Thal_MDm_Lt	12.0 (58.5)	113.8 ± 309.6	1.0 (9.0)	35.2 ± 128.7	378	**0.004**
	Thal_MDm_Rt	31,831.0 (2,778.0)	31,942.1 ± 2,605.1	33,260.0 (4,063.0)	32,526.4 ± 3,307.1	541	0.314

*AV, anteroventral; Habenula_Lt, left habenula; Habenula_Rt, right habenula; IQR, interquartile range; MDD, major depressive disorder; NC, normal control; ROI, region of interest; Thal_MDm_Lt, left magnocellular portion of the mediodorsal thalamus; Thal_MDm_Rt, right magnocellular portion of the mediodorsal thalamus; VA, ventral anterior. Statistical analysis was performed using the Mann–Whitney U-test. The number in bold indicates statistical significance after Bonferroni correction (Bonferroni-adjusted significant p-value level < 0.0083)*.

### Interhemispheric Difference of the WT of the Tracts From the Habenula to the Mediodorsal Thalamus

**Figure 3:**
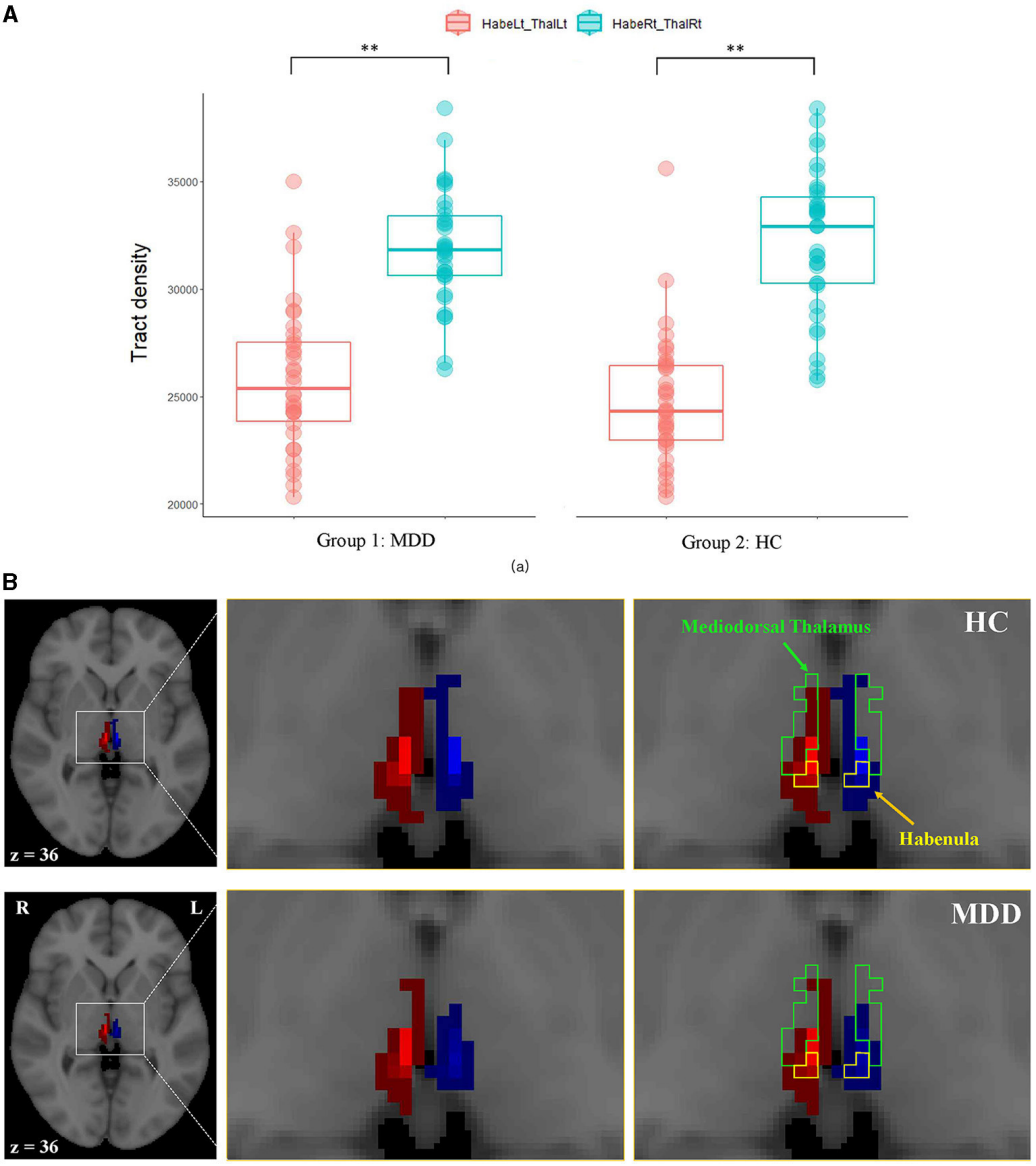
The comparison of ipsilateral tracts from the mediodorsal thalamus to the habenula between hemispheres. **(A)** Box plots of the tract densities between hemispheres in the MDD and NC groups, **(B)** a representative ipsilateral fiber connection in an MDD (bottom) and an NC (top) participant. Fiber connections between the right habenula and the right thalamus are shown in red, and fiber connections between the left habenula and the left thalamus are shown in blue. The green and yellow boundaries represent regions of the mediodorsal thalamus and the habenula, respectively. Lt, left; MDD, major depressive disorder; NC, normal control; Rt, right. **Indicates statistical significance (*p* < 0.001).

The left habenula seed had the most connections with the left mediodorsal thalamus, with a WT value of 25,817 and 24,983 in the MDD and NC groups, respectively (Table 2). The corresponding WTr of this left ipsilateral tract was ~50% in both groups (Table 3). The right habenula had the most connections with the right mediodorsal thalamus, and the WT values were 31,942 and 32,526 in the MDD and NC groups, respectively (Table 2). The corresponding WTr of this right ipsilateral tract was ~80% (Table 3). The WTr of the right ipsilateral tract was significantly greater than that of the left ipsilateral tract in both the MDD (79.9% [right] vs. 51.6% [left], 6,745 ± 728 [WT mean difference], *p* < 0.001) and NC (81.3% [right] vs. 50.0% [left], 7,901 ± 729 [WT mean difference], *p* < 0.001) groups (Table 3). A greater WTr in the right ipsilateral tract than in the left tract is also shown in Figure 3.

**Table 3:** The interhemispheric difference of the waytotal of the tracts from the habenula to the mediodorsal thalamus.

**Group**	**Seed ROI—Target ROI**	**WTr (%) Mean (± SD)**	**WT mean difference (± SD)**	**Statistics**	
				***t***	***p***
MDD	Habenula_Lt—Thal_MDm_Lt	51.6 (± 6.6)	6,745 (± 728)	−9.26	**<0.001**
	Habenula_Rt—Thal_MDm_Rt	79.9 (± 6.5)			
NC	Habenula_Lt—Thal_MDm_Lt	50.0 (± 6.0)	7,901 (± 729)	−10.83	**<0.001**
	Habenula_Rt—Thal_MDm_Rt	81.3 (± 8.3)			

*The statistical analysis was performed using a paired t-test. Habenula_Lt, left habenula; Habenula_Rt, right habenula; MDD, major depressive disorder; NC, normal control; ROI, region of interest; SD, standard deviation; Thal_MDm_Lt, left magnocellular portion of the mediodorsal thalamus; Thal_MDm_Rt, right magnocellular portion of the mediodorsal thalamus; WT, waytotal; WTr, waytotal ratio. P-values marked with bold indicate statistically significant differences*.

### Correlation Among the Number of Tracts in the Right Habenula-Left Mediodorsal Thalamus Pair and Clinical Characteristics in MDD Group

In the partial correlation analysis controlling for age among the right habenula-left mediodorsal thalamus pair, which showed significant differences in the number of tracts between the groups and several clinical variables, only duration of antidepressant use was positively correlated with the right habenula-left mediodorsal thalamus track (*r* = 0.398, *p* = 0.022).

## Discussion

The results of this study showed a thicker right habenula-left mediodorsal thalamus tract in the patients with MDD than in the NCs. On the other hand, there was no difference between the groups in the other tracts between the thalamus and the habenula. In addition, there was a higher right ipsilateral thalamo-habenular tract ratio (right thalamus-right habenula > left thalamus-left habenula) in both patients with MDD and NCs.

In this study, the number of right habenula-left mediodorsal thalamus tracts was greater in patients with MDD than in NCs. To the best of our knowledge, this finding on the connection between the thalamus and habenula in depression is novel, especially in human studies. In a previous study, treatment non-responders showed lower fractional anisotropy in the right habenula afferent fibers compared to responders, and the authors of the study suggested that decreased input to the habenula caused a reduction of neurons/gray matter (45). In a resting state fMRI study, the habenula showed significant connectivity with the thalamus; however, the authors did not report differential connectivity in this tract between participants with low and high subclinical depression scores (46). Animal studies using mice reported that the TCF7L2 gene, known as a risk factor for schizophrenia and autism (47), affected the connectivity and cell clustering of the thalamo-habenular region (48). Since this is a cross-sectional study, we are unable to determine the cause of the higher number of right habenula-left mediodorsal thalamus tracts in patients with MDD than in NCs. However, it can be assumed that more tracts are formed to compensate for deficits in the functional link between the thalamus and the habenula.

The habenula and the dorsomedial thalamic nucleus act as part of the extended limbic system, affecting the limbic system and playing a role as a center for emotions (49, 50), which may be related to depression (51). The habenula acts as a critical neuroanatomical hub between the forebrain and midbrain regions that regulate mood, motivation, and social behavior (52). The dorsomedial thalamic nuclei serve as a primary cortical transmit for the limbic system, offering significant connections to the prefrontal cortex (44). Therefore, the impaired function of the dorsomedial thalamus-habenula tract may be involved in negative emotions and decreased motivation.

Limbic systems such as the hypothalamus, basal forebrain, and thalamus are known as major regions that input neural signals to the habenula (18). However, in our study, most fibers departing from the habenula ended in the thalamus (about 50 and 80% for the left and right ipsilateral connections, respectively, in Table 3). In particular, the medial habenula is known as an input to the interpeduncular nucleus (IPN) from which signals are projected into the thalamus; thus, the fiber connections between the habenula and thalamus probably occur through the habenula-IPN-thalamus circuit (53–55). However, the evidence should be further examined.

This study also showed that the WTr of the right ipsilateral tract between the thalamus and habenula was significantly greater in both the MDD and NC groups. Numerous studies and experiments have identified asymmetrical structural circuits or functional laterality of the habenula in vertebrates. In a zebrafish experiment, the dorsal medial habenula was larger on the left side than on the right side, and the dorsal lateral habenula was larger on the right side than on the left side (56, 57). Although asymmetry or lateralization of the mammalian habenula had not been described until recently, in a recent human brain imaging study using high-resolution cardiac-gated resting state imaging, the right habenula showed greater functional connectivity with the substantia nigra and ventral tegmental area than did the left habenula and the left habenula showed greater functional connectivity with the parahippocampus than did the right habenula (32). In that study, the habenula was functionally connected to the thalamus, as previously found in a non-human study (32, 58). However, unlike our study, in that study there was no asymmetrical functional connectivity with the thalamus as both the left and right habenula seed regions showed a similar degree of positive correlation with the bilateral thalamus (32). Further studies examining functional connectivity and using DTI in other subjects are needed to clarify the asymmetry of the thalamus-habenula tract.

In the partial correlation analysis, the number of tracks increased as the duration of antidepressant use increased. This result suggests that the use of antidepressants is related to track compensation. However, this should be further explored by studies explicitly designed to examine this, such as prospective rather than cross-sectional studies.

To the best of our knowledge, our finding of a higher number of right habenula-left mediodorsal thalamus tracts in patients with MDD compared to NCs is novel. We also showed an interhemispheric asymmetry of the tract between the thalamus and habenula in both patients with MDD and NCs. These findings suggest that MDD could be related with the disintegration of the left thalamus-right habenula tract and an increased number of tracts as a compensational mechanism. The average duration of depression in this study was not very long (about 5 years), and most of the patients with MDD had received treatment for depression, so the number of tracks might have increased due to the compensational mechanism. However, if depression is prolonged or the severity of depression is extreme, it is possible that the number of tracks will decrease (59). Considering the limitations of this cross-sectional study, studies with larger samples and a longitudinal study design will be needed in order to investigate the temporal change of this relationship and determine the role of the thalamus-habenula tracts and the neural mechanism of MDD. This study had another limitation. Although the DTI parameters used in this study (i.e., *b* = 900 s/mm^2^) meet the standard of the research in this field (38), *b* = 1,000 s/mm^2^ might be better to obtain an appropriate signal-to-noise ratio. In addition, we expect further studies investigating connectivity in more brain regions and samples. These efforts will help identify the neurobiological evidence of MDD.

## Data Availability Statement

The raw data supporting the conclusions of this article will be made available by the authors, without undue reservation.

## Ethics Statement

All participants provided written informed consent to participate in the study. This study was approved by the Institutional Review Board (IRB No. GDIRB2018-005) of the Gil Medical Center. The patients/participants provided their written informed consent to participate in this study.

## Author Contributions

S-GK and C-KK: conceptualization. S-EC, K-SN, and S-GK: data acquisition. NK, C-KK, and S-GK: analysis. S-EC, NK, C-KK, K-SN, and S-GK: writing, review, and editing. All authors approved the final version to be published and no other individuals not listed as authors have made substantial contributions to the paper.

## Conflict of Interest

The authors declare that the research was conducted in the absence of any commercial or financial relationships that could be construed as a potential conflict of interest.

## Publisher's Note

All claims expressed in this article are solely those of the authors and do not necessarily represent those of their affiliated organizations, or those of the publisher, the editors and the reviewers. Any product that may be evaluated in this article, or claim that may be made by its manufacturer, is not guaranteed or endorsed by the publisher.

## Supplementary Material

Supplementary Figure 1 Segmented habenula, indicated with a red, overlaid on a T1 template image. **(A)** Axial, coronal, and sagittal images for indicating the segmented habenula. **(B)** Consecutive axial images covering the habenular region.
