# Supplementary figures and images for: Thalamo-Habenular Connection Differences Between Patients With Major Depressive Disorder and Normal Controls
Source: Front Psychiatry. 2021 Sep 1;12:699416. doi: 10.3389/fpsyt.2021.699416 (PMC8440934; doi:10.3389/fpsyt.2021.699416)

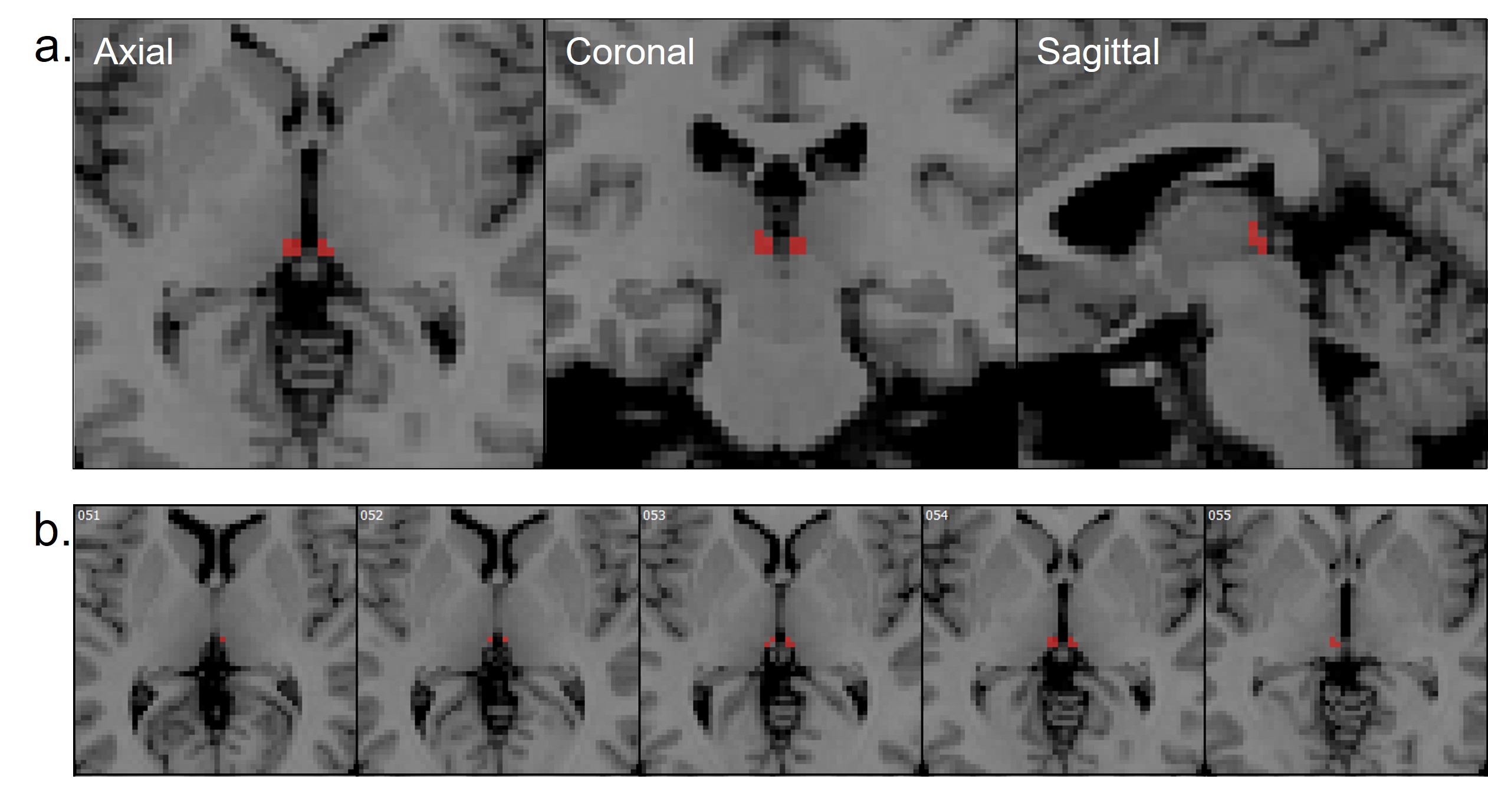

Supplement: Supplementary Figure 1 — Segmented habenula, indicated with a red, overlaid on a T1 template image. (A) Axial, coronal, and sagittal images for indicating the segmented habenula. (B) Consecutive axial images covering the habenular region. [file Image_1.JPEG]
